# Prevalence and Associated-Factors for *Entamoeba gingivalis* in Adolescents in Southeastern Iran by Culture and PCR, 2017

**Published:** 2020-02

**Authors:** Maryam SHARIFI, Fatemeh JAHANIMOGHADAM, Zahra BABAEI, Mohammad Ali MOHAMMADI, Fatemeh SHARIFI, Nima HATAMI, Masumeh DANESH, Parnian POURESLAMI, Hamidreza POURESLAMI

**Affiliations:** 1. Department of Pediatric Dentistry, School of Dentistry, Kerman University of Medical Sciences, Kerman, Iran; 2. Oral and Dental Diseases Research Center, Kerman University of Medical Sciences, Kerman, Iran; 3. Kerman Social Determinants on Oral Health Research Center, Kerman University of Medical Sciences, Kerman, Iran; 4. Department of Medical Parasitology and Mycology, School of Medicine, Kerman University of Medical Sciences, Kerman, Iran; 5. Leishmaniasis Research Center, Kerman University of Medical Sciences, Kerman, Iran; 6. Research Center for Hydatid Disease in Iran, Kerman University of Medical Sciences, Kerman, Iran; 7. Department of Pharmaceutics Research Center, Institute of Neuropharmacology, Kerman University of Medical Sciences, Kerman, Iran; 8. Department of Endodontic, School of Dentistry, Kerman University of Medical Sciences, Kerman, Iran

**Keywords:** *Entamoeba gingivalis*, Prevalence, Adolescents, Associated factors, Iran

## Abstract

**Background:**

This study aimed to identify the prevalence and potential factors associated with *Entamoeba gingivalis* in adolescents in the city of Kerman, southeastern Iran, 2017.

**Methods:**

In this descriptive cross-sectional study, 315 adolescents (mean age; 15 yr) consisting of 189 males and 126 females were randomly selected. For each adolescent, two specimens were collected for culturing and examination by polymerase chain reaction (PCR). Univariate and multivariate logistic regression models were performed to explore any association with demographic and clinical variables.

**Results:**

The prevalence of *E. gingivalis* was 11.7%. Totally, 30 (15.9%) males and 7 (5.6%) females were infected with *E. gingivalis*. The rate of infection in males was 2.8 times higher than that in females (*P*<0.001). Statistical analysis identified 4 major factors including sex (OR=4.12, *P*<0.001), gingival index with severe inflammation (OR = 50, *P*<0.001), *Candida* spp. infection (OR=4.41, *P*<0.001) and decay-missing-filled teeth [DMFT (OR=3.27, *P*<0.001)]. In contrast to the aforementioned factors, adolescents with history of antibiotic consumption were significantly protected from *E. gingivalis* infection (OR= 3.24, *P*<0.001). Culture media detected 9.2% (n= 29), whilst PCR identified 11.4% (n= 36) of infection.

**Conclusion:**

The present findings clearly demonstrate a positive association between *E. gingivalis* and distinct demographic and clinical risk determinants. Therefore, dental practitioners and health surveillance personnel should be aware of these confounding factors to rigorously detect and critically manage oral health issues in school-age children in order to prevent or at least minimize the eventual periodontal complications in later life.

## Introduction

*Entamoeba gingivalis* is a cosmopolitan anaerobic amoebic protozoan wildly distributed in oral cavity of humans (1). Trophozoite has variable size range of 10–35μm. The amoeba has no cyst in its life cycle; therefore, it is transmitted either directly via direct contacts, mainly by kissing or indirectly through trophozoite-contaminated food, toothpicks, gum or other utensils (2). This opportunistic organism inhabits the gums and in the area surrounding the teeth including the spaces between teeth cavities, along the gingival fringes of gums, dental tartar, necrotic mucosa around the teeth and also in the gingival pockets (3).

*E. gingivalis,* aside from its contribution to bad smelling of the mouth, could be an indicator of oral health status and it is generally considered as an oral commensal but reports show that it displays a pathogenic potential associated with gingivitis (4–6) in children and eventually with periodontal diseases in later life, especially in minority groups such as immunocompromised patients (7, 8). The majority of the periodontal disturbances in higher age groups commonly have their stem earlier in childhood life (9–11).

Epidemiological reports demonstrated variable prevalence rates of *E. gingivalis* infection among the children (8, 14). Its occurrence may vary according to age, presence of gingivitis, periodontitis and immunosuppressive conditions (7, 14, 15). School children are extremely susceptible to a broad range of gingival infections such as bacteria and protozoa (9, 10, 16). Gingivitis with varying degrees of severity is a universal finding and a common phenomenon in children. Obviously, there are controversies concerning its pathogenicity characteristic as this amoeba is commonly found in healthy children and with gingivitis as well (17).

Therefore, the present study aimed to explore the prevalence and associated-factors for *E. gingivalis* in a group of adolescents in southeastern of Iran by culture and PCR in 2017.

## Materials and Methods

### Ethical statement

This project was granted approval by the Ethics Committees of Kerman University of Medical Sciences (project no. 95000230, Ethics no. IR.KMU.REC1395.409).

All studied school children, authorities and teachers and if required their parents, were well informed of the aims and procedures and those willingly interested to participate. Those who signed the written informed consent form were included in the study.

### Design and population

This study was performed as a cross-sectional and analytical survey between January and June 2018 at School of Medicine, Kerman University of Medical Sciences, Kerman, eastern Iran. The adolescents were randomly selected from four elementary schools consisting of two males and two females. Each adolescent was subjected to a thorough examination of the oral cavity. For each individual, a structured questionnaire was completed and all demographic and clinical characteristics were recorded.

### Inclusion and exclusion criteria

Overall 382 school children were examined and of which 67 individuals were excluded due to various reasons. Of the children examined, 315 were deemed eligible for enrolment in the study.

### Sample collection

For each adolescent, two specimens were collected by scraping debris along the gingival line using a sterile periodontal curette (3, 18, 19). One sample was inoculated into a tube containing sterile physiological saline solution and stored at −20 °C for further PCR examination. The other specimen was cultured in a biphasic medium tube containing horse serum slant overlaid, 6 ml Ringer’s solution (HSr Medium), 200 μl of rice starch (5 mg/ml) and 2% penicillin/ streptomycin (20). The inoculated tubes were labeled and promptly transported to the Medical Parasitology Diagnostic Laboratory at School of Medicine. The cultured tubes were incubated at 35.5 °C in digital incubator and examined two times at 48 h intervals by a light microscope.

### Diagnosis of the isolates

**Fig. 1:**
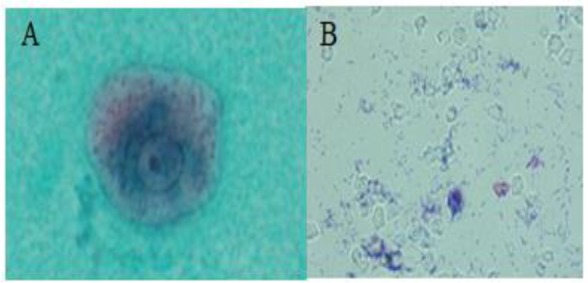
*Entamoeba gingivalis,* trichrome-stained (A) and *Trichomonas tenax*, Giemsa-stained (B)

The detection of *E. gingivalis* was performed following growth of the trophozoite in the culture media. A clean slide was prepared and small drop of liquid medium alongside the solid phase was placed on the slide and the wet smear preparation was thoroughly examined visually by a light microscope (×400 magnifications). In addition, for some isolates, portion of the samples were transferred into polyvinyl alcohol fixative and stained by trichrome staining technique and by Giemsa (21) (Fig. 1 A&B).

### Molecular identification DNA extraction

DNA was directly extracted from *E. gingivalis* trophozoite obtained from the culture media. Briefly the organism was washed three times in physiological saline solution (3000 rpm for 5 min, pH; 7.2). DNA was extracted by proteinase K using the High Pure Template Purification Kit (Roche, Germany) based on the Kit’s instruction.

### Polymerase chain reaction (PCR)

The extracted DNA was used for amplification of the ribosomal RNA of *E. gingivalis* and *Trichomonas tenax* by polymerase chain reaction (PCR) with self-designed specific primers based on the previous records of ribosomal DNA in nucleotide database of NCBI. A 454-bp fragment of the SrRNA gene, amplified by specific forward (5′-GCGCATTTCGAACAGGAATGTAGA-3′) and reverse (5′-CAAAGCCTTTTCAATAGTATCTTCATTCA-3′) primers for *E. gingivalis* was used. Also, a 496-bp fragment of 18S ribosomal RNA gene and precise forward (5′- ATGACCAG-TTCCATCGATGCCATTC -3′) and reverse (5′- CTCCAAAGATTCTGCCACTAACAAG -3′) primers for *T*. *tenax.* All primers were designed with primer 3 online program (22) and checked for specific amplification with Primer-BLAST tools (23) and confirmed with positive known isolates. PCR was performed in 50μl using 3.5 mM MgCl2, 250 mM of each of the dNTPs, 40 pmol of each primer, 2 U Taq polymerase, and 4 mL (20–100 ng/mL) of the DNA template, under the following thermal profile: 5 min at 94 °C as an initial denaturation step, followed by 35 cycles of 30 sec at 94 °C, 30 sec at 58 °C, 60 sec at 72 °C, and a final extension step of 10 min at 72 °C. The amplicons were electrophoresed on 1% (w/v) agarose gel. DNA of reference strains of *E. gingivalis* and *T. tenax* were used as positive and distilled water as negative controls. The DNA ladder was Gene Ruler 100 bp (Roche, Germany).

### Statistical analysis

Data were entered into SPSS ver. 20 (Chicago, IL, USA) and the significance of statistical difference between proportion (χ^2^) was determined. In this study, the associated factors which might have roles with amoebic infection as defined by odds ratios were calculated. An odds ratio (OR) is a measure of association between causative agent (*E. gingivalis)* and demographic or clinical characteristics compared to the odds of those with no infection (control group). Initially, the univariate logistic regression model was used to evaluate each variable individually. Then those variables with *P*-value <0.2 were analyzed by multivariate logistic regression method by backward stepwise manner to exclude the effect of confounding factors. The significance level was considered at *P*<0.05.

## Results

### Demographic and Clinical characteristics

Overall, 315 children in four adolescent schools consisting of two males (n=189) and two females (n=126) (Table 1) were randomly selected (mean age; 15 and range; 13–16 yr old).

**Table 1:** Baseline characteristics and infection rate of *Entamoeba gingivalis* in adolescents in suburban areas of Kerman city by culture and PCR, 2017

***Characteristics***	***Total***		***Infected***	
Variable	No.	%	No.	%
Male	189	60.0	30	15.9^*^
Female	126	40.0	7	5.6
Total	315	100	37	11.7

* *P*<0.001

**Fig. 2:**
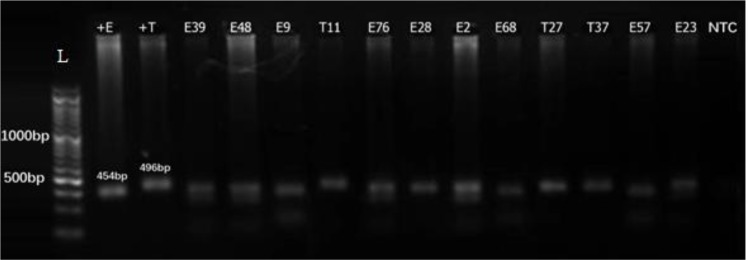
Agarose gel electrophoresis of the representative *Entamoeba gingivalis* and *Trichmonas tenax* isolates obtained from adolescents in suburban areas of Kerman city by culture and PCR, 2017. L: DNA size marker, 100 bp; NTC: negative control (distilled water); +E: positive control (*Entamoeba gingivalis,* 454 bp); +T: positive control (*Trichomonas tenax,* 496 bp); E39, 48, 9, 76, 28, 2, 68, 57 and 23 *E. gingivalis;* T11, 27, 37 *T. tenax* (clinical isolates).

They were interviewed and clinically examined for oral health status and for *E. gingivalis* and *T. tenax* infection by direct smear microscopy, culture media and molecular identification using PCR technique. In general, 11.7% (n=37) of the adolescents were infected with *E. gingivalis* including 30 (15.9%) males and 7 (5.6%) females. The proportion of the infection rate in males was 2.8 times higher than that in female children (*P*<0.001). In the present investigation 7 cases (2.2%) of *T. tenax* were also identified. Due to the limited number of infections and consequent inconclusive analysis, this protozoan was not included in the study. Culture media detected 9.2% (n=29), whilst PCR identified 11.4% (n= 36) (Fig. 2).

### Associated-factors analysis

The univariate logistic regression analysis demonstrated 5 main associated-factors (Table 2) including; namely sex (OR=4.12, CI=1.8–9.6, *P*<0.001), history of antibiotic consumption (OR=3.243, CI=1.68–6.27, *P*<0.001), gingival index with category of severe inflammation (OR=50, CI= 4.53–551.41, *P*<0.001), *Candida* species co-infection (OR= 4.41, CI = 2.04–9.26, *P*<0.001) and DMFT (OR=3.26, CI=2.35–4.5, *P*≤0.001) were significantly associated with *E. gingivalis* infection rate in adolescents*.* In contrast to the aforementioned factors, adolescents with history of antibiotic consumption were significantly protected from the *E. gingivalis* infection (OR=3.24, CI=1.68–6.27, *P*<0.001) as detected by only univariate model. The multivariate model confirmed only 3 major risk determinants (Table 3) consisting of gingival index with severe inflammatory reaction (OR=30.14, CI=1.10–823.31, *P*<0.04), *Candida* spp. co-infection (OR= 3.74, CI=1.32–10.56, *P*<0.013) and DMFT (OR= 3.27, CI= 2.30–4.66, *P*<0.001). Factors which showed a negative association with infection by univariate and multivariate model included parents’ occupation and history of systemic diseases (Table 4).

**Table 2:** Associated-factors for *Entamoeba gingivalis* in adolescents analyzed by univariate logistic model in suburban areas of Kerman city by culture and PCR methods, 2017

***P value***	***95% CI***	***OR***	***Infected No (%)***	***Total***		***Characteristic***
< 0.001	1.8–9.6	4.12	30 (81.1)	189	Male	Sex
		1	7 (18.9)	126	Female	
		1	16 (43.2)	133	Yes	History of antibiotic use
< 0.001	1.68–6.27	3.243	21 (56.8)	182	No	
		1	1 (2.7)	23	No inflammation	
	0.12–10.29	1.12	5 (13.5)	72	Mild inflammation	
	0.29–17.77	2.26	21 (56.8)	207	Moderate inflammation	Gingival Index
< 0.001	4.53–551.41	50	10 (27.0)	13	Sever inflammation	
< 0.001	2.04–9.26	4.41	29 (78.4)	263	Yes	*Candida* spp*.*
		1	8 (21.6)	52	No	
< 0.001	2.37–4.5	3.26		315	Yes	DMFT^*^
				0	No	

* Decay-missing and filled teeth

**Table 3:** Associated-factors for *Entamoeba gingivalis* in adolescents analyzed by multivariate model in suburban areas of Kerman city by culture and PCR methods, 2017

***P value***	***95% CI***	***OR***	***Infected No (%)***	***Total***		***Characteristic***
		1	1 (2.7)	17	No inflammation	
	0.92–30.06	1.66	5 (13.5)	87	Mild inflammation	Gingival Index
	0.3–52.17	3.46	21 (56.8)	196	Moderate inflammation	
<0.04	1.10–823.31	30.14	10 (27)	15	Sever inflammation	
<0.013	1.328–10.56	3.744	29 (78.4)	171	Yes	*Candida* spp.
		1	8 (21.6)	144	No	
<0.001	2.30–4.66	3.27	37 (100)	315	Yes	DMFT^*^
		1		0	No	

* Decay-missing and filled teeth

**Table 4:** Non-associated factors for *Entamoeba gingivalis* in adolescents in suburban areas of Kerman city by culture and PCR methods, 2017

***Characteristic***		***Infected No (%)***	***Total***	***OR***	***95% CI***	**P *value***
Fathers’ job	Unemployed	1 (5.5)	23	0	0	1
	Free job	27 (63.8)	195	1.28	0.63–2.61	0.5
	Employee	9 (30.6)	97	1		
Mothers’ job	Unemployed	0 (0)	15	1.06	0.30–3.76	0.92
	Free job	34 (91.9)	214	0	0	1
	Employee	3 (8.1)	86	1		
History of systemic diseases	Yes	2 (5.4)	33	1.13		0.88
	No	35 (94.6)	282	1	0.24–5.26	

## Discussion

In this study, the prevalence rate of *E. gingivalis* was fairly high. This findings might be negatively related to how school children perceive their oral health affairs. Oral hygiene is a key component of overall health status. The impact of the parasitic agents in periodontium pathology is still scarce and controversial (24). However, not only bacteria remain as the etiological agents of the periodontal diseases, but also parasites could be the cause (4–6). Periodontal diseases including gingivitis and periodontitis are the impact of disruption between the host-oral microbiome involvements. These diseases are caused by diverse and complex multiple factors (6, 24–26). The infection rate was significantly higher in male than female group. One possible explanation for male elementary school children showed higher rate of infection might be related to the epidemiological and behavioral differences. On the other hand, females are more disciplined and sensitive to oral health issues compared with male school children. The exact association between the sex and the risk of amoeba is complex and requires further investigation (27).

According to our data overall 11.7% of the adolescents showed infection with *E. gingivalis,* whilst a prevalence rate of 18% was reported from Shiraz (17). In comparison with our findings, an infection rate of 41.7% with *E. gingivalis* was reported in patient referred to the Faculty of Dentistry in Tehran, demonstrating a significantly higher prevalence rate compared with the data found by the present study (28). In contrast, in Ahvaz, only 0.5% of the examined individuals were infected with this amoeba, performing a lower frequency relative to our finding (29). On the other hand, in Ahvaz, 15.7% of the age range 6–14 yr old were infected with *E. gingivalis* and simultaneously they had also gingivitis (30). Due to presence of various factors including age, gender, socioeconomic characteristics, poor oral health conditions, such comparisons are inconclusive (6). Moreover, several other associated-factors seem to have some roles in prevalence of infection with *E. gingivalis* entails periodontal tissue damage, poor oral health condition, tooth loss, dental caries, low educational level, cigarette smoking, drugs and food (5, 31).

The amoeba possesses the capacity to be involved in sever gingival inflammatory reactions. Overall, the infection rate of *E. gingivalis* in adolescent school children was higher among those with severe gingival index compared to the healthy control group. This result is consistent with that found by Luszczak et al (5). *E. gingivalis* is able to alter the leukocyte metabolism by excessive production of elastase, a mechanism by which enabling the amoeba to induce gingival bleeding and severe inflammation. In addition, the role of this amoeba in pathophysiological changes of periodontium has been reported (25, 26). This amoeba is capable of producing substances to optimize the anaerobic condition for their long survival. Moreover, the high infection rate of this protozoan in the mouth cavity is well-associated with poor oral hygiene and concluded this amoeba is common among subjects with low standard of living conditions (32) and also in patients with human immunodeficiency virus type-1 (HIV-1) (7, 15, 33). Other demographic and clinical factors including age, gender, cigarette smoking and presence of periodontal diseases could play a role in prevalence of this amoeba.

The use of broad-spectrum drugs likewise has potential effect in providing fertile grounds for *Candida* species co-infection in this study. *C. albicans* is concurrently found to be enhanced in association with numerous microorganisms including bacteria (34). The interactions involved between the organisms in co-infection are the complexity and diversity of this mutual relationship. One of the main reasons for little attention to this issue would be difficult to understand such association.

Decay- missing- filled teeth (DMFT) in children particularly in adolescent schools is an important oral and public health issue. The US Dentists for Disease Control and Prevention have reported that tooth decay is the most prevalent chronic childhood disease, over 5 times more frequent than asthma in children, 5–17 yr old (35). One of the main reasons for subjects who have DMFT is the creation of a suitable space for dwelling and replication of the microorganisms (15, 36). This organism tends to be involved in cases of poor oral hygiene and poor periodontal health (14, 16, 37).

The present study was carried out during the cold months of the year when common cold was a prevalent event in aggregated school-aged children. Therefore, the consumption of local or systemic drugs such as antibiotics (amoxicillin and tetracycline) and other anti-infective agents with broad spectrum of action against various parasitic and microbial organisms is common phenomenon (38). Metronidazole is the drug of choice for treatment of all intestinal parasites including amoebae, *Giardia* and trichomonads and also for wide ranges of anaerobic bacteria (39). The use of such broad action drugs could have residual or major impact on this amoeba and related bacterial microflora (3, 6, 19).

The diagnosis of parasitic infections in oral cavity is still based on direct microscopic examination of debris and tissue scrapings. However, this method has low sensitivity. Culturing of the organism along with DNA-based PCR methods (29 vs. 36 cases, respectively) are highly accurate with higher sensitivity power (6); although, in this study comparison of the methods was not the purpose.

## Conclusion

The present findings clearly demonstrated a positive association between *E. gingivalis* and demographic and clinical risk determinants. Knowledge of the major risk factors for *E. gingivalis* infection is essential in improving public and oral health strategies. Therefore, dental practitioners and health surveillance personnel should be aware of these confounding factors to rigorously detect and critically manage oral health issues in school-age children in order to prevent or at least minimize the eventual periodontal complications in later life.

## Ethical considerations

Ethical issues (including plagiarism, informed consent, misconduct, data fabrication and/or falsification, double publication and/or submission, redundancy, etc.) have been completely observed by the authors.
